# Selection of Nest Material and Summer Nest Location by the Hazel Dormouse (*Muscardinus avellanarius*) in the Bidstrup Forests, Denmark

**DOI:** 10.3390/biology12010139

**Published:** 2023-01-16

**Authors:** Heidi Holm Hansen, Sara Sofie Bertelsen, Cino Pertoldi, Sussie Pagh, Helle Vilhelmsen

**Affiliations:** 1 Department of Chemistry and Bioscience, Aalborg University, 9220 Aalborg, Denmark; 2 Aalborg Zoo, 9000 Aalborg, Denmark; 3 Independent Researcher, Bontvedvej 13, 5700 Svendborg, Denmark

**Keywords:** vegetation, understorey, habitat, Jacob’s selectivity index

## Abstract

**Simple Summary:**

The hazel dormouse is a threatened species and knowledge about its ecology is vital for conservation purposes. It constructs summer nests directly in the vegetation, in tree hollows, or in nest boxes. The availability of nest materials and vegetation coverage may affect the likelihood of finding hazel dormice at a location. The aim of this study is: (1) To investigate the preferences of hazel dormice for nesting materials today compared to four decades ago. (2) To investigate hazel dormice preferences for vegetation coverage at nest sites. Beech, grass, and bark are the most important nest materials in both period A: 1980–1985 and period B: 2019–2020, although the nests from period A contained more beech and less oak compared to nests from period B. Therefore, ensuring the availability of these preferred nest materials is important in conservation efforts for the species in Denmark. Coverage of shrubs above 2 m is important for nest site selection. Beech and high understorey (2–8 m) may be crucial in the conservation management of hazel dormice in Denmark.

**Abstract:**

Hazel dormice (*Muscardinus avellanarius*) construct summer nests for resting and breeding. The nests are built directly in the vegetation, in tree hollows, or in nest boxes. The availability of nest materials and vegetation coverage may affect the likelihood of finding hazel dormice at a location. The aim of the study is: (1) To investigate the preferences of hazel dormice for nesting materials today compared to four decades ago. (2) To investigate hazel dormice preferences for vegetation coverage at nest sites. In total, 148 hazel dormouse summer nests from the Bidstrup forests in Zealand (Denmark), were analysed. Of these, 82 were collected in the period A: 1980–1985 and 66 were collected in B: 2019–2020. In total 26 different nest materials were found. Beech was the major nest material in both periods, and Jacob’s selectivity index indicates that beech is selected for as nesting material and that hazel dormice may travel to collect beech leaves. Nests from period A contained more beech (W = 1521, *p* < 0.05) and less oak (W = 1304, *p* < 0.01) compared to nests from period B. Vegetation analysis showed that coverage of shrubs higher than 2 m above ground (W = 1.5, *p* = 0.07) may be of great importance for hazel dormice.

## 1. Introduction

The hazel dormouse (*Muscardinus avellanarius*) is widely distributed across Europe and considered as Least Concern on the IUCN redlist [1,2]. However, in large parts of its range, it is declining and considered threatened as well as being red-listed in many countries, including Denmark [1,2,3,4]. For this reason, the hazel dormouse has been included in Appendix III of the Bern Convention and also listed in Annex IV of the EU Habitats Directive. This obliges the participating countries to protect the hazel dormouse and its habitats [5,6]. In Denmark, the distribution of the hazel dormouse is restricted to only a few isolated locations on Funen, Zealand, and Jutland [7]. Hazel dormice have disappeared from several locations in Denmark [8]. The hazel dormouse is an arboreal animal, living in deciduous or mixed deciduous–coniferous forests [1]. It avoids the ground level of the forest and is, therefore, dependent on continuous vegetation for movement [9,10,11]. Its preferred habitat includes vegetation at different successional stages, clearings, and a rich understorey [1]. Furthermore, it prefers a habitat with high plant diversity, providing food throughout its active period [12,13,14]. The hazel dormouse is mainly threatened because of habitat loss, fragmentation of habitats and unfavourable forest management practices [7,15,16,17]. Previous hazel dormouse locations may have lost favourable vegetation structures to support resilient hazel dormouse populations. Very few hazel dormouse habitats are considered of high enough quality to maintain stable populations, which means that many populations are at risk of local extinction [7]. Detailed knowledge about the preferred nesting material and vegetation structure of hazel dormice would be decisive in order to prevent a further decline in the Danish hazel dormouse population.

In conservation management of rare and threatened species, such as the hazel dormouse, it is important to have a broad knowledge of the species ecology and resource requirements [9,18]. Furthermore, requirements can differ depending on the time of the year and it is necessary to cover all seasons to achieve a successful conservation effort [19]. An important part of the ecology of many species is nest construction. It is observed in a wide range of taxa, including mammals, birds, and invertebrates [20,21,22,23]. The hazel dormouse builds both summer and winter nests, which are very similar in structure [24,25,26,27]. Usually, nests are categorized into four different types, as suggested by Wachtendorf (1951): foliar nests, built of leaves from trees and bushes possibly woven together by a few strands of grass; grassy nests, solely containing grass or shredded bark; layered nests, which have an outer layer of leaves and an inner layer of grass or bark; and mixed nests, which also consist of leaves and grass or bark, but intermixed throughout the nest.

The aim of the study is: (1) To investigate the preferences of hazel dormice for nesting materials today compared to four decades ago. (2) To investigate the preferences of the hazel dormouse for vegetation coverage of the nest location. Conservation strategies for Danish hazel dormouse habitats are discussed.

## 2. Materials and Methods

### 2.1. Study Site

**Figure 1:**
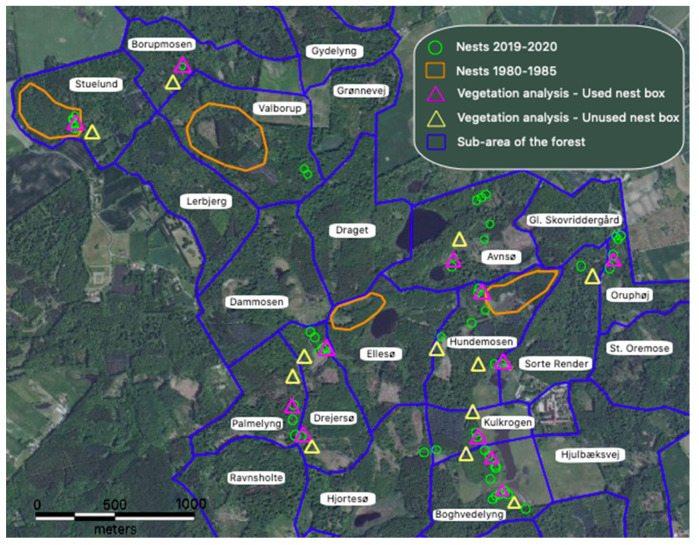
Study area in the Bidstrup forests, where blue lines indicate sub-areas of the forest. Green circles indicate locations of nests from 2019 to 2020. Orange linings indicate the locations of nests from 1980 to 1985, as no coordinates were available. Pink triangles indicate locations of vegetation analysis around used nest boxes, while yellow triangles indicate locations of vegetation analysis around unused nest boxes.

The study was conducted in the Bidstrup forests, which are located on Zealand in Denmark (55°33′27″ N 11°52′39″ E) (Figure 1). The climate in Denmark is coastal temperate. The Bidstrup forests are several hundred years old and forestry has always been a part of the area. While the production of wood is still important in the Bidstrup forests, a focus on protection of the nature, and especially hazel dormice, has led to the felling of coniferous trees, to create clearings with self-seeding of deciduous trees. The investigated part of the forest is protected as a Natura2000 area [28]. The forest is approximately 1000 ha and contains 500 nest boxes for hazel dormice, placed in locations presumed to be optimal for hazel dormice after the Danish habitat standards [7]. In 2018, the first 300 nest boxes were placed, and in 2019 and 2020 an additional 100 nest boxes were placed each year. The area contains a mixture of various deciduous and coniferous trees, dominated by beech (*Fagus sylvatica*) in most of the forest and oak (*Quercus* spp.) in small parts of the forest. Other main tree species growing in the area are birch (*Betula* spp.), linden (*Tilia cordata*), sycamore (*Acer pseudoplatanus*), ash (*Fraxinus excelsior*), hornbeam (*Carpinus betulus*), Norway spruce (*Picea abiea*), and Sitka spruce (*Picea sitchensis*). The main understorey species include hazel (*Corylus avellana*), rowan (*Sorbus aucuparia*), blackberry (*Rubus fruticosus*), and raspberry (*Rubus idaeus*).

### 2.2. Nest Data

The data set consists of a total of 148 hazel dormouse summer nests. One was excluded as it contained dead offspring of unidentified *Apodemus* spp., which was impossible to separate entirely from the nest materials. Another one was excluded as it consisted entirely of ash seed coats, which are the remains of food storage by *Apodemus* spp. Of these, 82 were collected in the years from 1980 to 1985 by Vilhelmsen (1989) [29], and 66 were collected in 2019 and 2020 by a group of volunteers. The locations where nests have been found can be seen in Figure 1. All nests were collected from nest boxes. Nest boxes are placed in locations with hazel dormouse activity, documented by findings of natural nests in the vegetation, during the dormant winter period of the dormice, as part of the management program in Denmark. The nest box is a wooden box constructed as a bird box, with the entrance hole facing the tree trunk. The box is placed in trees 1–1.5 m above ground. The exact location, with coordinates, nest box number, and date of collection, was noted.

In Denmark, the nest of the hazel dormouse can at first glance be confused with the nests of e.g., the harvest mouse (*Micromys minutus*), the Eurasian wren (*Troglodytes troglodytes*), and the chiffchaff (*Phyloscopus colybita*). Therefore, all nests were examined in the laboratory and distinguished as hazel dormouse nests following the differences in shape, size and material described by Vilhelmsen, 2011 [30]. In the laboratory, the nests were photographed and weighed. Furthermore, the nest type was noted as foliar (F), layered (L), mixed (M), or grassy (G) according to the definitions by Wachtendorf (1951) [24]. After this, the nest was deconstructed and all nest material was categorized as leaves, grass, bark, moss, etc. Only leaves of trees and bushes were taxonomically determined to genus. A reference collection of plant material, as well as the standard Danish flora, were used for the taxonomic determination [31]. Thirty nests contained nuts. Nuts were excluded from the total weight of these nests, as they are not considered as nest material. The nests from 1980 to 1985 have been stored in boxes at room temperature for 36–41 years. As the recently collected nests were stored at −18 °C immediately after collection, they had to be dried. This was carried out after deconstruction at 60 °C for 48 h. After 48 h, the dry weight of each component of the nest was measured.

### 2.3. Vegetation Data

The methodology used for analysing the vegetation was based on the technical document for forest habitats made by NOVANA [32]. Vegetation data were collected in 24 spots around the investigated forest area. Sample plots were placed, centred around a nest box, half of which have been inhabited by a hazel dormouse and half of which have not (Figure 1). Each plot consisted of two circles, an inner circle with a diameter of 5 m and an outer circle with a diameter of 15 m. In the 5 m circle, a complete species list with species relevant for the hazel dormouse was conducted. This includes trees, bushes, and species under the nettle genus, mosses and ferns, as these are known to be used by the hazel dormouse for nest construction [24,25,26,27,29]. Furthermore, the coverage of trees and bushes above and below 2 m, grasses, moss, ferns and the specific herbs mentioned, are registered. Any clearings, paths, water covered areas, tree stumps, trunks and dead standing trees are registered as well. In the 15 m circle, an additional species list was conducted, including only species not found in the 5 m circle.

### 2.4. Data Analysis

All analyses and figures were achieved using the statistical software Rstudio (Version 1.3.1073). All dry weights of nest materials were transformed into proportions of the total nest weight. To find changes in the selection of nest material over time, the Mann–Whitney test was used to find significant differences in the median proportion of nest materials of nests from 1980 to 1985 and nests from 2019 to 2020. Furthermore, a chi-squared test was used to investigate changes in the choice of nest type between the two periods. A linear regression model was carried out to investigate if hazel dormice use the most accessible nest materials. The mean proportions of nest materials were plotted against the mean proportion of those materials within 5 m of the nest boxes. Selection of nest materials were tested by comparing the median proportion of materials used in the nests with the median proportion of materials found within 5 m of the nest boxes. The differences were tested for significance using a pairwise Mann–Whitney test. To investigate the selectivity of nest materials found in both nests and vegetation analysis from nest sites with findings of nests, a selectivity index (Jacob’s *D*) was calculated. The selectivity index follows the formula $D=\frac{r-p}{r+p-2rp}$ where *r* represents the proportion of nest material in the nests and *p* represents the proportion of available nest material in the vegetation surrounding the nest site. The index was calculated for each location and nest separately, using the Jacob electivity function of the electivity package in Rstudio [33]. The median Jacob’s index was plotted for each nest material with a 25–75% interquartile range. Mann–Whitney was used to compare vegetation around nest boxes with hazel dormouse nests and nest boxes without hazel dormouse nests.

## 3. Results

### 3.1. Nest Materials

In total, 82 summer nests from 1980 to 1985 and 66 from 2019 to 2020 were examined. Excluding unidentifiable materials, the total number of nest materials used was 18 in the period 1980–1985 and 22 in 2019–2020. The major components of the nests from 1980 to 1985 are beech leaves (48.26%), grass (21.11%), and bark (17.05%). The major components of the nests from 2019 to 2020 are beech leaves (34.99%), grass (17.27%), and oak leaves (11.89%) (Table 1). These nest materials also made up the heaviest part of the material in most nests (Table 1). The number of materials used per nest ranged from 1 to 11 (n = 148, M = 5, IQ = 4–6). The median proportion of a single material in a nest ranges from 0.06 (0.04–0.3) for seed coma to 40.06 (25.8–57.4) for beech (Table 1). All four nest types (F-, G-, L-, and M nests) were represented in both time periods (Table 2). The chi-square test showed significant differences between the choice of nest type in the two periods (X2 = 10.6, df = 3, *p* < 0.05).

### 3.2. Changes in Selection of Nest Materials over Time

**Figure 2:**
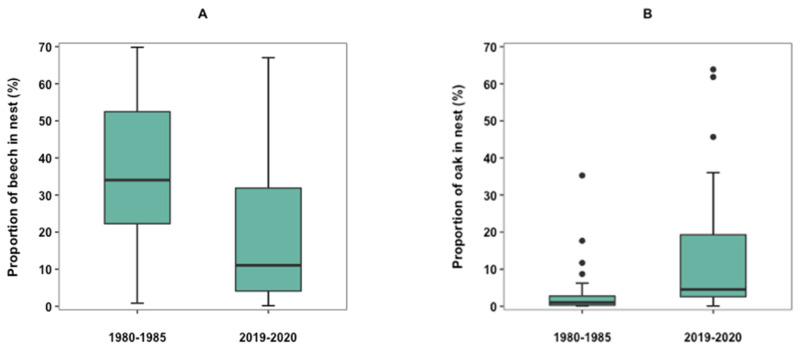
Boxplots exhibiting the proportion of beech (**A**) and oak (**B**) in nests from 1980 to 1985 (n = 82) and nests from 2019 to 2020 (n = 66). Boxes represent the 25 to 75% interquartile range, and the lines are the median values. Error bars extend upward from the third quartile to the maximum and the other extends downward from the first quartile to the minimum. Outliers are represented by dots.

Beech leaves were used significantly more in the nests from 1980 to 1985 (W = 1521, *p* < 0.05), whereas oak leaves were used significantly more in the nests from 2019 to 2020 (W = 1304, *p* < 0.01) (Figure 2A,B). All other nest materials in the nests from 1980 to 1985 were not significantly different from the nest materials of the nests from 2019 to 2020 (Table 1). Seven nest materials were found in nests from 2019 to 2020, but not in nests from 1980 to 1985. These are alder (*Alnus glutinosa*), field maple (*Acer campestre*), seed coma, hazel, honeysuckle (*Lonicera spp.*), hornbeam, and linden. Three nest materials were found in nests from 1980 to 1985, but not in nests from 2019 to 2020. These are cypress (*Cupressus sempervirens*), blackberry, and elm (*Ulmus glabra*), and the last two only occurred in one nest each (Table 1).

### 3.3. Selection of Nest Materials

**Figure 3:**
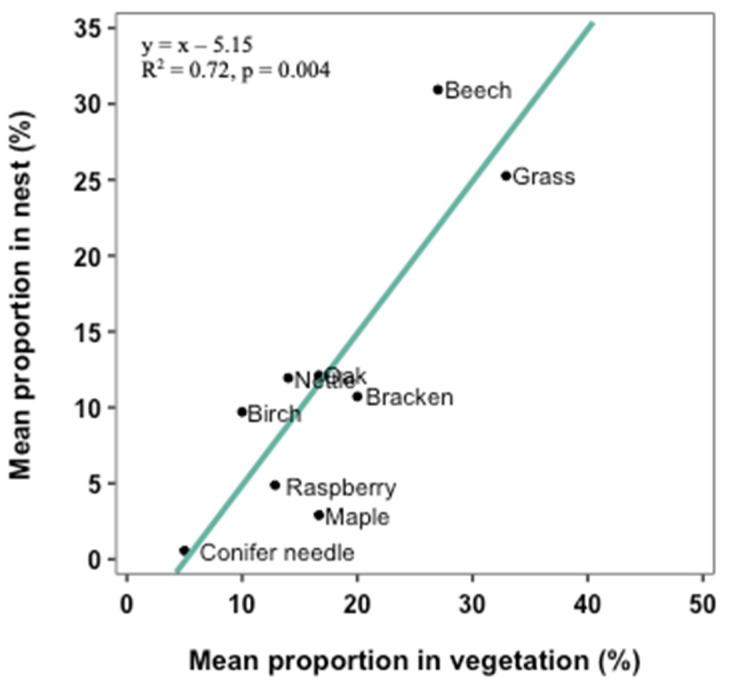
Relationship between availability of nine plant materials and their use in hazel dormouse summer nests. These plant materials are beech, grass, oak, nettle, bracken, birch, raspberry, maple, and conifer needle. The proportion of material in nests is plotted against the proportion of material in the vegetation within 5 m of the nest box. A linear green tendency line is plotted as well with the equation y = 1x − 5.15 and a fit of R^2^ = 0.72.

The total number of plants found in the vegetation analysis was 30. Of these, nine did not appear as nest material in the nests. These were Fern, Buckthorn (*Rhamnus cathartica*), Rush (*Juncaceae* spp.), Willow (*Salix* spp.), Elder (*Sambucus nigra*), Cherry (*Prunus* spp.), Bulrush (*Typha* spp.), Currant (*Ribes* spp.), and Barberry (*Berberidinae* spp.). Furthermore, elm was used as a nest material, but it did not appear within the 15 m of the vegetation analysis. Twelve materials were found both in the nests and in the vegetation within 5 m of the nest boxes. The proportions of three nest materials were significantly higher in the vegetation analysis within 5 m around the nest boxes compared to the proportion in the nests. These nest materials were hazel leaves (W = 24, *p* < 0.05), honeysuckle leaves (W = 32, *p* < 0.01), and moss (W = 54, *p* < 0.05). It is concluded that these materials are not used in general as nest materials. The materials used as nest material show a positive linear tendency (R^2^ = 0.72, *p* = 0.004), meaning that the larger the availability of the nest material is, the more it is used in the nests (Figure 3).

**Figure 4:**
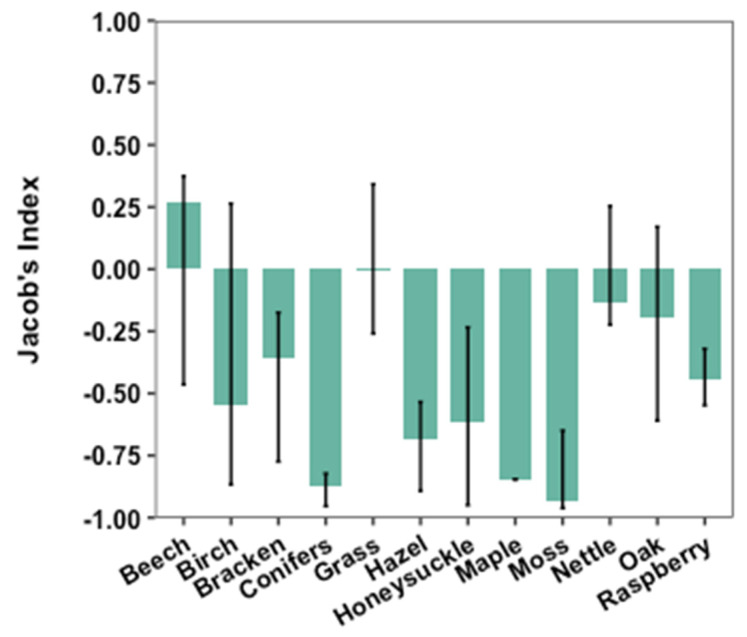
Selectivity indices (Jacob’s index of selection) for 12 nest materials occurring in both hazel dormouse nests and the surrounding vegetation. Materials with an index between 0 and 1 are selected for, while materials with an index between 0 and −1 are avoided. Materials close to 0 are considered neutral, meaning that they are neither selected nor avoided. The lines through the bars show the interquartile range (25–75%).

To visualise the possible preferences of nest material, Jacob’s index of selection was plotted for the 12 nest materials found both in the nests and in the vegetation within 5 m of the nest boxes (Figure 4). Most materials have negative selectivity indices, meaning that they are abundant in the vegetation surrounding the nest boxes and are readily available to cover the need of these materials for nest construction (Figure 4). Grass has a selectivity index close to zero, as it occurs in appropriate amounts in the vegetation to cover the need for nest construction. Beech is the only material with a positive selectivity index, meaning that the hazel dormouse travels further to collect this material if it is not readily available (Figure 4).

### 3.4. Nest Site Selection

**Figure 5:**
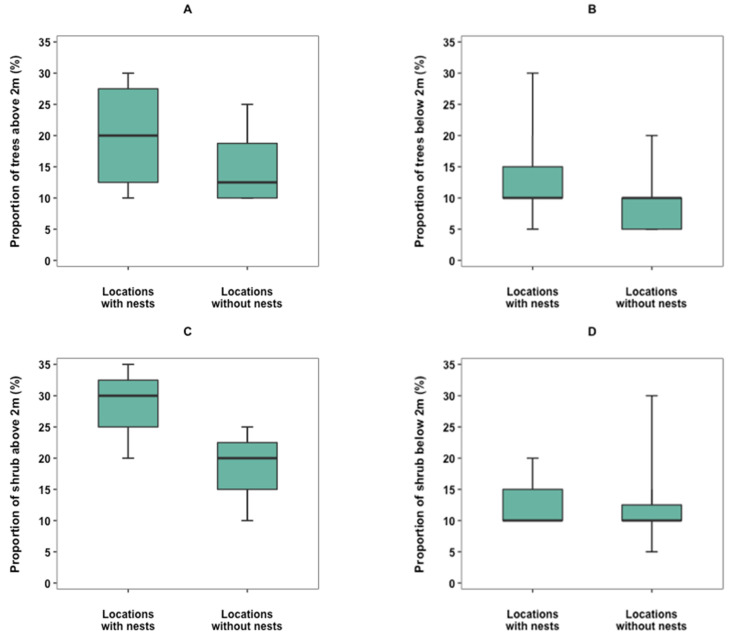
Boxplots exhibiting the proportion of (**A**) trees above 2 m, (**B**) trees below 2 m, (**C**) shrubs above 2 m, and (**D**) shrubs below 2 m within 5 m of the nest box. Locations with findings of hazel dormouse nests (n = 12) are compared to locations without findings of hazel dormouse nests (n = 12). Boxes represent the 25 to 75% interquartile range, and the lines are the median values. Error bars extend upward from the third quartile to the maximum and the other extends downward from the first quartile to the minimum.

Differences were found between the vegetation at sites with findings of hazel dormouse nests compared to sites without findings. The coverage of trees above 2 m is larger at locations with nests than locations without nests, although the difference is not significant (W = 65, *p* = 0.71, n.s.) (Figure 5A). The same applies to the proportion of trees below 2 m (W = 22, *p* = 0.29, n.s.) (Figure 5B) and the proportion of shrubs below 2 m (W = 46, *p* = 0.96, n.s.) (Figure 5C). The proportion of shrubs above 2 m is higher at locations with nests, compared to locations without nests (Figure 5D), and the difference is at the boundary of significance (W = 1.5, *p* = 0.07, n.s.). The presence of each plant species found in the investigations of the vegetation, as well as the species richness, did not differ significantly (*p* > 0.05).

## 4. Discussion

### 4.1. Nest Materials

Nest materials are an important resource for hazel dormice and the knowledge of possible preferences is beneficial in the conservation management of the species. Several studies have been conducted on the nest material of the hazel dormouse in England, Ukraine, Slovakia, Russia, Belgium, Moldova, Germany, Austria, and Lithuania [1,25,26,27,34,35,36]. The main nest materials, grass and leaves, can be found in most habitats. However, the plant species used as nest material might vary across regions and countries, and is dependent on the composition of the plant community [1]. The total number of nest materials in the present study were higher compared to studies by Čanády (2015) [26], Bracewell and Downs (2017) [35], and Gubert et al. (2022) [27], who found 11, 18, and 11 materials, respectively. In this study, beech leaves were the nest material used most often, which corresponds to studies from Germany, by Möckel (1988, as cited in [1]), and Austria by Henze and Gepp (2004, as cited in [1]), who found that beech leaves were preferred as a nest material. Studies by Lozan (1970, as cited in [1]) and Airapetyants (1983, as cited in [1]) found that beech leaves were also a common nest material in Moldova. Based on the similarities, it appears that beech leaves are a preferred material for the construction of hazel dormouse nests in several regions of its distributional range. Henze and Gepp (2004, as cited in [1]) found that oak leaves were too hard and therefore not used for nest construction. The same was found by Verbeylen et al. (2017) [36], where tougher leaves from, e.g., ivy, hawthorn, oak, and beech were used less frequently. However, in Moldova, both Lozan (1970, as cited in [1]) and Airapetyants (1983, as cited in [1]) found nests to most often include oak leaves. In this study, oak leaves occurred in more than half of the nests, indicating a preference for this material. In Germany, Möckel (1988, as cited [1]) found oak, but also rowan, birch, and maple to be used in locations where beech leaves were rare or absent. In Lithuania, beech trees are absent from hazel dormouse habitats [1]. Here, Juškaitis (1997) [37] found that hazel leaves are often used as a nest material, while beech leaves are not. Furthermore, hazel leaves are used in high proportions in England, according to Morris et al. (1990) [34] and Bracewell and Downs (2017) [35], and also in Belgium, according to Verbeylen et al. (2017) [36]. In a study from Ukraine by Zaytseva (2006) [25], they found wood material, feathers, wool and anthropogenic materials in form of thread. With the exception of wool, all these materials were found in this study as well. However, they primarily appeared in hazel dormouse nests built on top of birds’ nests. This apparently results in the dormouse reusing available material from the bird nest in its own nest construction. Two studies from England by Morris et al. (1990) [34] and Bracewell and Downs (2017) [35] found honeysuckle bark to be the most frequently used material. In this study, bark was the third most used material, but it was not taxonomically identified to species level. It is possible that honeysuckle bark plays a more important role for nest construction than this study exhibits. The differences in preferred nest materials across different regions indicate that the hazel dormouse has preferences for certain nest materials, but also has a high plasticity if the preferred materials are not readily available.

In a study by Čanády (2015) [26], they found that M-nests were the most apparent nest type (73.0%), followed by L- (15.7%), G- (9.0%), and finally F-nests (2.3%). In this study, F-nests were the second most apparent type, followed by L- and G-nests in both time periods. F-nests being frequent in nest boxes was also found in studies by Juškaitis (1997) [37] and Zaytseva (2006) [25]. The study by Čanády (2015) [26] was carried out on natural nests in the vegetation, while this study investigates nests from nest boxes. This suggests that the hazel dormouse chooses which nest type to build depending on external conditions. In nest boxes, the walls are able to support the fragile structure of the F-nests; hence, this nest type might be preferred. In comparison, nests built in the vegetation might need grass or bark, especially the long strings from honeysuckle bark, woven into the nest to uphold the structure, leading to a low proportion of F-nests. In a study by Gubert et al. (2022) [27], hibernation nests were primarily built as L-nests (42.4%), followed by G- (30.3%), F- (18.2%), and M-nests (9.1%). As a rule, L-nests are built for breeding [1]. However, the low proportion of M-nests as hibernation nests compared to the summer nests of this study might be a reflection of M-nests also being used as breeding nests and L-nests also being used for hibernation.

### 4.2. Selection of Nest Materials

The proportions of three nest materials were significantly larger in the vegetation compared to the nests. Hazel, especially, constituted large proportions of the surrounding vegetation but was rarely used in nest constructions, which was also observed by Zaytseva (2006) [25]. In Lithuania, England, and Belgium, however, hazel leaves are often used for nest construction, which emphasises the regional differences in the selection of nest material [35,36,37]. Additionally, moss appeared at most nest sites, but was rarely used in nests, which corresponds to investigations on hibernation nests by Gubert et al. (2022) [27]. Moss is a frequent plant in woods and is, therefore, available and used for nest construction by many birds [38]. Hazel dormice, however, do not seem to prefer moss for nest construction, and use it primarily when building their nest on top of birds’ nests. Moss grows in locations with a certain moisture level, which indicates that the selection of nest site is affected by the microclimate. Although hazel rarely occurs in nests, it frequently occurs in the vegetation analysis; hence, it may act as an important understorey vegetation for hazel dormice and the nuts as an important food source during autumn. With the exception of hazel, materials have a tendency of being used more frequently when they are more accessible in the vegetation. The same observation was made by Gubert et al. (2022) [27], who found that all used materials were present within 3 m of the nest. Verbeylen et al. (2017) [36] also found that both leaves from the tree supporting the nest box and from adjacent trees were most commonly used for nest construction. This suggests that hazel dormice primarily use available materials close to the nest. Additionally, Zaytseva (2006) [25] found that the choice of nest material was dependent on the abundance and availability in the vegetation, but also individual preferences. This supports the findings in this study of nine materials in 1980–1985 and eight materials in 2019–2020 appearing in less than 10% of the nests, indicating individual preference. As an example, blackberry only appears in one nest, which could be a result of an individual preference for the material, and it does not seem to be an important nest material for hazel dormice in general. Elm was used in one nest, but did not appear in any vegetation analysis. Dormice may travel further for this particular nest material. However, the material was only used in one nest and the vegetation analysis did not cover all nest boxes with findings of nests. Therefore, it does not seem to be a material of preference, but more likely it is a matter of availability at the given nest site or individual preference.

In a study by Juškaitis (2014) [1], the results of several authors are summarised and it is hypothesised that nest material differs in different regions depending on the plant composition of the habitat. In this study, several plants appeared in the vegetation analysis but not in the nests. This indicates that hazel dormice have preferences in nest material, as certain species are avoided. This is supported by Gubert et al. (2022) [27], who found 11 out of 31 available materials in the nests. In this study, beech and grass appear in large proportions in the nests, indicating a preference for these materials. As the selectivity index of beech is positive, it confirms beech to be a preferred nest material, which the hazel dormouse travels further to collect if it is not apparent in the surrounding vegetation. Bracewell and Downs (2017) [35] investigated the distance travelled to collect different nest materials and found a significant difference between materials. Here, certain materials were collected from over 10 m away, including oak leaves, beech leaves and honeysuckle bark [35]. Furthermore, honeysuckle bark was the material appearing as the most preferred, as hazel dormice travelled up to 50 m for this particular nest material [35]. These findings support this study in beech leaves being preferred, and the possibility of honeysuckle bark being more important as a nest material than was shown by this study. The fact that the selection index confirms the preference for beech and avoidance of hazel as nest material could be due to beech leaves being smaller and, therefore, easier to handle. New beech leaves, especially, may be softer and more pliable than leaves from hazel. Moreover, Jacob’s selectivity index exhibits that most nest materials cover enough of the surrounding area to support the need for these in nest construction.

### 4.3. Changes in the Selection of Nest Materials and Nest Type over Time

To the best of our knowledge, no studies have previously investigated if the materials of hazel dormouse summer nests have changed over time. In this study, we see a significant change in the selection of nest type. Although M-nests were the preferred type in both periods, they made up twice as large a proportion of the nests in the years 2019–2020 as they did in 1980–1985. As the nest material seem to vary depending on availability in the surrounding vegetation, it is possible that nest material has changed in cases where the vegetation has changed. If the hazel dormouse is specific in its selection of nest material, changes in the vegetation over time would not determine the nest material but rather nest site selection. In 1980–1985, the woods contained 410 ha of beech and 161 ha of oak [29]. Beech occurred in 92.3% of the locations analysed, compared to only 75% of locations analysed in this study. The smaller proportion of available beech could explain the lower occurrence of beech in the nests from 2019 to 2020. However, beech is still today the most dominant tree species in the Bidstrup forests, while oak is less available. This does not reflect the decreased proportion of beech in the nests, and the explanation could be the positioning of the nest boxes. As part of the management plan for the hazel dormouse in the Bidstrup forests, the thinning of corridors has been carried out in 2018, 2019, and 2020. Here, large shadowing beech trees have been felled to make room for oak trees and understorey vegetation in form of hazel, raspberry, blackberry, and hawthorn [39]. As these are the areas where nest boxes are placed, oak might be more available to hazel dormice today, compared to 1980–1985. This may explain the higher proportion of oak in nests from 2019 to 2020 than nests from 1980 to 1985. This further contributes to the hypothesis that hazel dormice are flexible in their choice of nesting material and to a large extent use what is available.

### 4.4. Nest Site Selection

The proportion of the recorded plant species did not differ significantly between sites with nest boxes used by hazel dormice and sites with unused nest boxes. Additionally, species richness was similar in vegetation analysis of sites with used and unused nest boxes. This is in contrast with studies from several countries, including Sweden [40], England [9], Lithuania [41], and also a study by Mortensen et al. (2022) [42] from another part of Denmark. These studies found that species richness is one of the most important features for nest site selection of the hazel dormouse. In this study, the species list of the vegetation analysis is concentrated on possible nest materials. Furthermore, the Bidstrup forests are an old forest area which includes a high species diversity across most of the forest [28]. Nest boxes are also placed in locations with known dormouse activity and vegetation known to be preferred by hazel dormice. For this reason, species diversity might be high at both sites with and without findings of hazel dormouse nests and, therefore, might not show any significant differences in this study. Species diversity was the second major discriminant of dormouse density in a study by Bright and Morris (1990) [9]. It is suggested that species diversity is important in a hazel dormouse habitat, as it provides a continuum of food sources throughout the season [9]. The dietary composition of the hazel dormouse changes throughout its active period, depending on availability of food items [43]. As it is an opportunistic feeder, it is suggested by Goodwin et al. (2020) [44] that habitat conservation should include increasing and maintaining the abundance and distribution of flower- and fruit-bearing shrubs and trees as well as invertebrate populations at a fine spatial scale. Securing high plant and invertebrate species diversity in hazel dormouse habitats could, therefore, be important, although it is not shown by the results of this study. The hazel dormouse forages in a range of approximately 100 m and one individual builds more than one nest [1,34]. Furthermore, telemetry observations have shown that nest sites do not correspond to foraging areas [10]. This suggests that nest site selection is led by characteristics other than species diversity. Nest site selection might be affected by interspecific competition. In the Bidstrup forests, six nest boxes have been found to hold a bird nest at the end of the season when the nest boxes were checked. Of the hazel dormouse nests from this study, 19 were built on top of birds’ nests. This indicates that nest boxes might be selected on the basis of some of the same variables by both birds and dormice. In England, long-term volunteer records were examined by Williams et al. (2013) [45], who found a negative correlation between nest boxes containing birds’ nests and dormice. This implies possible competition or mutual exclusion. *Parus* spp. are the primary birds nesting in dormouse nest boxes [1]. Morris et al. (1990) [34] found that the activities of *Parus* spp. were most intense early in the year, and normally they disappeared by the time dormice needed the nest boxes. This fits well with the findings of dormice nests on top of birds’ nests in this study, and it suggests that no interspecific competition with birds is apparent in terms of nest site selection. Nuts, especially acorns and hazel nuts, were found in 30 of the 148 nests. Furthermore, it was observed that some nests contained large pools of excrements and one nest contained offspring of unidentified *Apodemus* spp. Hazel dormice are not known to store nuts for later consumption, hence, these are all signs of *Apodemus* spp. [1]. In a study by Marsh and Morris (2000) [46] it was found that, besides the hazel dormouse, the yellow-necked mouse (*Apodemus flavicollis*) used nest boxes more than any other small mammal. However, they found litters to be uncommon, suggesting that they rarely used nest boxes for breeding [46]. Hazel dormouse nests are very attractive to other species. Wood mice (*Apodemus sylvaticus*) have been found to take over hazel dormouse nests and occasionally even kill hazel dormice to do so [1]. This implies that *Apodemus* spp. are possible competitors for dormice nest boxes. Vegetation structures have been investigated, as well, as they have been shown to facilitate nest site selection. The importance of understorey vegetation in a hazel dormouse habitat, especially, has been proven by Bright and Morris (1990) [9]. In this study, the coverage of shrubs below 2 m of height does not differ between sites with findings of dormouse nests, compared to sites without findings of dormouse nests. However, considering the importance of the understorey vegetation structures in a hazel dormouse habitat, it contributes to the future maintenance of an optimal habitat as it grows. In a study by Panchetti et al. (2007) [10], hazel dormice preferred nest boxes in areas with a high density of understorey, and the density of the understorey was more relevant for nest site selection than species diversity. In a study by Verbeylen et al. (2017) [36], radio-collared hazel dormice were tracked and the preferred height for placement of the nest, both in the vegetation, nest boxes, and nest tubes, was between 0 and 2 m. This shows that the height for the placement of nest boxes in this study, and in Denmark in general, seems optimal, as they are placed around a height of 1.5 m.

In this study, certain differences were found in vegetation structures between sites with and without findings of hazel dormouse nests. At locations with findings of hazel dormouse nests, the coverage of shrubs above 2 m of height is larger than at locations without findings of nests, although the difference was not significant. Shrubs above 2 m ranged approximately between 2 and 8 m in height and were part of the understorey vegetation. As these parameters cover a larger proportion of locations with hazel dormouse nests, it appears to be an important feature in nest site selection. It indicates that young vegetation is important in an optimal hazel dormouse habitat, securing routeways and possibly spatially separating the overlapping dietary niche with *Apodemus* spp. This corresponds to a study from England by Goodwin et al. (2018) [47], where hazel dormice preferred mid-height vegetation between 5 and 10 m. Securing these mid-successional habitats requires a dynamic optimum of felling and regeneration in the conservation management of hazel dormouse populations [47]. Furthermore, Goodwin et al. (2018) [47] found that yew, rowan, and hazel were preferred during ranging. Juškaitis et al. (2013) [41] investigated vegetation structures 900 m^2^ around each nest, and found hazel to be the most important shrub species in the understorey. In this study, hazel was not found to be an important nest material; however, it occurred in approximately half of the vegetation analysis. This indicates that hazel is an important species, providing dense shrub vegetation with arboreal pathways and connectivity between nest boxes and higher trees. As vegetation analysis with regard to nest site selection is usually conducted around nests, understorey vegetation structures appear as the most important factor in a hazel dormouse habitat. However, it seems that when investigating a larger area, as in Juškaitis et al. (2013) [41], both species diversity and density of understorey vegetation are key factors in providing the optimal habitat for hazel dormice. In this study, coverage of trees both above and below 2 m are larger at locations with findings of nests than at locations without. Although not significant, the results indicates that the vegetation density is larger at locations with findings of nests. The habitat selection of hazel dormice in Denmark has been investigated by Mortensen et al. (2022) [42], showing that dormice strongly select sites with high vegetation density of woody plants and high species richness. This emphasizes the results of this study, showing the importance of vegetation structures around 2–8 m. Securing this in the conservation management of the hazel dormouse would possibly improve habitat quality and the carrying capacity for hazel dormice [42]. Most investigated nest box locations had a large number of felled trees laying on the ground. This creates better penetration of sunlight to the forest floor, which improves the coverage of understorey species. This is beneficial for the hazel dormouse in the long run; however, until sufficient regrowth of the understorey, it might cause dormice to avoid using those nest boxes. At some locations, the position of the felled trees imitated understorey vegetation as the branches overlapped with trees and shrubs, creating arboreal routeways for easy movement, which makes these nest boxes more attractive to hazel dormice. This is confirmed when looking at a study by Juškaitis et al. (2013) [41], who found that the connectivity of nest boxes with the surrounding trees was a significant predictor of nest site selection. This indicates that the positioning of felled trees might be a relevant strategy in conservation management to ensure access to the nest boxes, when regenerating habitats to make transitional stages of understorey vegetation less challenging.

## 5. Conclusions

Hazel dormice use a wide range of nest materials in accordance to their availability in the surrounding vegetation; however, they do seem to have a preference for beech. Beech, grass, and bark are the most important nest materials. Therefore, ensuring the availability of these preferred nest materials is important in conservation efforts of the species in Denmark. The selection of nest box was not found to depend on species richness or the proportion of certain plant species. However, the coverage of shrubs above 2 m is important for nest site selection and may reduce competition between the hazel dormouse and especially *Apodemus* spp., which are also known to climb in the lower vegetation. Beech and high understorey (around 2–8 m) may be crucial in the conservation management of hazel dormice in Denmark.

## Figures and Tables

**Table 1:** Nest materials found in hazel dormouse summer nests from the period 1980 to 1985 and 2019 to 2020. IQ = interquartile range. The difference in median proportion of nest material between periods was tested using Mann–Whitney. Asterisk (*) indicate significant values.

Nest Material (No. of Nests with Material)	1980–1985			2019–2020			Difference in Median Proportion of Nest Material between Periods
	Proportion of Nests with Material (%)	Median % of Material in Nests (IQ Range)	Proportion of Nests with Material as Heaviest Component (%)	Proportion of Nests with Material (%)	Median % of Material in Nests (IQ Range)	Proportion of Nests with Material as Heaviest Component (%)	
Alder, *Alnus glutinosa* (n = 9)	-	-	-	13.43	6.96 (1.5–9.3)	4.48	-
Anthropogenic (n = 5)	6.10	0.29 (0.3–0.4)	-	1.49	1.42 (1.4–1.4)	-	W = 5, *p* = 0.24
Bark (n = 55)	67.07	8.82 (2.6–26.7)	14.63	43.28	15.32 (5.7–26.5)	7.46	W = 931, *p* = 0.21
Beech, *Fagus sylvatica* (n = 72)	87.80	40.06 (25.8-57.4)	51.22	86.57	18.40 (5.8–49.7)	38.81	W = 1521, *p* = 0.0053 *
Birch, *Betula* spp. (n = 5)	6.10	1.17 (1.0–3.1)	-	10.45	1.22 (0.5–8.6)	1.49	W = 18, *p* = 1
Blackberry, *Rubus fruticosus* (n = 1)	1.22	16.36 (16.4–16.4)	-	-	-	-	-
Bracken, *Pteridium aquilinum* (n = 20)	24.39	1.02 (0.2–19.5)	-	11.94	7.72 (2.4–16.0)	-	W = 109, *p* = 0.15
Branches (n = 41)	50.00	0.89 (0.5–1.9)	-	16.42	1.23 (0.6–1.4)	-	W = 232, *p* = 0.89
Conifer Needle, *Pinophyta* spp. (n = 55)	67.07	0.47 (0.2–1.2)	-	11.94	0.36 (0.1–1.0)	-	W = 176, *p* = 0.37
Cypress, *Cupressus sempervirens* (n = 2)	2.44	0.35 (0.2–0.5)	-	-	-	-	-
Elm, *Ulmus glabra* (n = 1)	1.22	12.14 (12.1–12.1)	-	-	-	-	-
Field Maple, *Acer campestre* (n = 9)	-	-	-	26.87	0.96 (0.8–6.0)	2.99	-
Grass, *Poaceae* (n = 59)	71.95	26.86 (5.8–64.1)	30.49	52.24	14.12 (5.3–38.4)	17.91	W = 883, *p* = 0.24
Hawthorn, *Crataegus monogyna* (n = 1)	1.22	0.74 (0.7–0.7)	-	5.97	1.85 (1.3–3.7)	-	W = 4, *p* = 0.29
Hazel, *Corylus avellana* (n = 6)	-	-	-	8.96	3.91 (1.9–5.6)	-	-
Honeysuckle, *Lonicera* spp. (n = 8)	-	-	-	11.94	7.55 (1.3–8.9)	1.49	-
Hornbeam, *Carpinus betulus* (n = 9)	-	-	-	13.43	8.54 (2.8–17.5)	2.99	-
Linden, *Tilia cordata* (n = 5)	-	-	-	7.46	10.59 (4.2–12.7)	-	-
Maple, *Acer pseudoplatanus* (n = 1)	1.22	12.87 (12.9–12.9)	-	1.49	2.91 (2.9–2.9)	-	W = 0, *p* = 1
Moss, *Bryophyta* (n = 39)	47.56	0.75 (0.3–5.0)	2.44	34.33	0.52 (0.3–3.2)	4.48	W = 452, *p* = 0.97
Nettle, *Urtica dioica* (n = 12)	14.63	4.30 (1.6–13.0)	-	7.46	10.17 (2.3–14.6)	1.49	W = 36, *p* = 0.56
Oak, *Quercus* spp. (n = 30)	36.59	0.99 (0.3–2.8)	1.22	80.60	4.52 (2.6–19.3)	11.94	W = 1304, *p* = 1.08 × 10^−5^ *
Raspberry, *Rubus idaeus* (n = 3)	3.66	1.19 (0.7–5.4)	-	5.97	4.78 (2.9–6.8)	-	W = 8, *p* = 0.60
Rowan, *Sorbus aucuparia* (n = 3)	3.66	1.31 (0.9–13.9)	-	25.37	5.44 (1.3–16.1)	2.99	W = 32, *p* = 0.53
Seed Coma (n = 3)	-	-	-	4.48	0.06 (0.04–0.3)	-	-

**Table 2:** Distribution of nest types in the periods 1980–1985 (n = 82) and 2019–2020 (n = 66). The chi-squared test shows a significant difference between selection of nest type in the two periods.

Nest Type	% of Nests from 1980 to 1985	% of Nests from 2019 to 2020
Mixed (M)	26.83	49.25
Foliar (F)	29.27	20.90
Layered (L)	28.05	20.90
Grass (G)	15.85	8.96
Chi-squared (X^2^)	X^2^ = 10.6, df = 3, *p* < 0.05	

## Data Availability

Not applicable.
